# Results from a 2020 field experiment encouraging voting by mail

**DOI:** 10.1073/pnas.2021022118

**Published:** 2021-01-19

**Authors:** Daniel J. Hopkins, Marc Meredith, Anjali Chainani, Nathaniel Olin, Tiffany Tse

**Affiliations:** ^a^Department of Political Science, University of Pennsylvania, Philadelphia, PA 19104;; ^b^Mayor’s Office, City of Philadelphia, Philadelphia, PA 19107

**Keywords:** voting by mail, field experiment, voter turnout, elections

## Abstract

The ability to cast a mail ballot can safeguard the franchise. However, because there are often additional procedural protections to ensure that a ballot cast in person counts, voting by mail can also jeopardize people’s ability to cast a recorded vote. An experiment carried out during the COVID-19 pandemic illustrates both forces. Philadelphia officials randomly sent 46,960 Philadelphia registrants postcards encouraging them to apply to vote by mail in the lead-up to the June 2020 primary election. While the intervention increased the likelihood a registrant cast a mail ballot by 0.4 percentage points (*P* = 0.017)—or 3%—many of these additional mail ballots counted only because a last-minute policy intervention allowed most mail ballots postmarked by Election Day to count.

The United States has long used absentee voting to protect the voting rights of people who find it difficult to vote at a polling place, such as military personnel and citizens with disabilities (1). Elections taking place since the COVID-19 pandemic demonstrate the benefit of mail ballots. Some who would have normally preferred to vote in person found that voting by mail allowed them to maintain social distancing (see also refs. 2 and 3). In response, many states increased the availability of mail ballots. Consequentially, the share of ballots cast by mail increased sixfold over prior elections in several states holding elections between April and June 2020 (4).

While increased access to mail ballots has helped protect voting rights, there are also concerns that their increased use could disenfranchise voters (5, 6). A mail ballot may be less likely to count than a ballot cast in person for multiple reasons: 1) Mail ballots may not be received in time, 2) mail ballots may have higher rates of clerical errors, and 3) the process of casting an in-person ballot may identify and rectify errors (4). The share of mail ballots affected by these issues likely increased during the COVID-19 pandemic. Partly, that is because people who would typically cast an in-person ballot are more likely to cast a problematic mail ballot than those experienced with voting by mail. Also, election officials are more likely to face issues distributing and tabulating mail ballots when there is an upsurge in mail ballots.

To better understand how access to mail balloting affected Americans’ ability to vote during the pandemic, we analyze a field experiment conducted in Philadelphia leading up to Pennsylvania’s 2 June 2020 primary election. Philadelphia had a stay-at-home order in place from 23 March until after the primary. Two weeks before the election, 46,960 randomly selected registrants received postcards with information about applying to vote by mail. This was the first statewide federal election held since Pennsylvania adopted universal access to mail ballots, so information about how to apply was particularly likely to increase awareness of voting by mail.

Previous field experiments show that facilitating mail ballot requests increases their use. Refs. 7 and 8 show that sending a registrant a prefilled mail-ballot application substantially increased the rate at which registrants requested mail ballots. Ref. 9 finds that mailing registrants a mail-ballot application and return envelope increased the use of mail balloting more than providing information about requesting a mail ballot (see also ref. 10).

Our results show that registrants receiving a postcard about mail balloting were at least as likely to successfully cast a ballot in the 2020 primary as those that did not, but that this occurred partly because of a late change in policy. Registrants receiving the postcard were about 0.5 percentage points more likely to request a mail ballot (*P* = 0.004), and about 0.4 percentage points more likely to return a mail ballot (*P* = 0.017), than registrants who did not. Roughly half of the additional mail ballots cast by registrants sent postcards were received after Election Day. While Pennsylvania state law specifies that mail ballots must be received by Election Day, issues with mail ballot distribution and large-scale protests following George Floyd’s death led Pennsylvania’s governor to order that ballots postmarked by Election Day be counted if received within a week of Election Day. While such estimates are noisy, the changes in in-person voting and overall turnout jointly suggest that 35% of the increased voting by mail came from substitution by people who would have otherwise voted in person.

## Experimental Design

We seek to answer three research questions. We are primarily interested in 1) whether postcards from local officials increase applications to vote by mail and secondarily 2) whether the postcards’ wording matters or 3) their effect is stronger among those who had received four postcards as part of a prior 2019 experiment. The University of Pennsylvania Institutional Review Board approved this study (832927).

The universe for this experiment consists of 935,745 registered voters. This represents a subset of the 1,048,575 registered voters in the universe for a previous mobilization experiment conducted by the City of Philadelphia and the City Commissioners prior to the 2019 municipal election.* The goal of the previous experiment was to see whether receiving multiple postcards over several months—including feedback on recipients’ vote history—fostered turnout via expectations of ongoing contact (11). Registered voters in the universe for the previous experiment were randomly assigned to receive flights of zero, two, or four postcards during the 2019 election cycle. The universe for this experiment excluded 88,807 people who were no longer registered voters in Philadelphia and roughly 24,000 people assigned to receive two postcards—an intermediate treatment—in the prior experiment. Because we sought to examine interactions between the two experiments, we tested whether there were any differences in the likelihood that people previously mailed four and zero postcards remained registered to vote. The absence of differential attrition enables us to straightforwardly estimate not only the main effects but also interactions between the 2019 and 2020 postcards.^†^

**Fig. 1.**
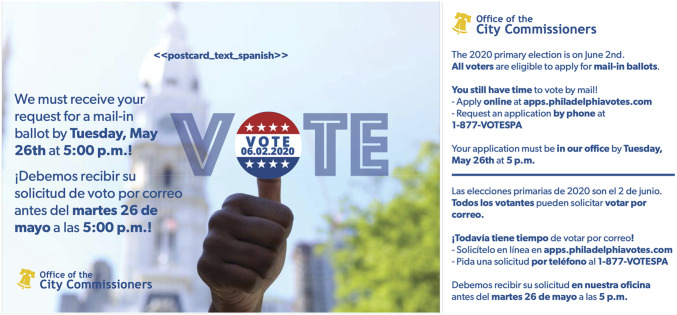
Front and back of sample treatment postcards.

Registrants in our experimental universe were sent one of two postcards about mail ballots or no postcard. The postcards conveyed information about the 26 May deadline to request a mail ballot and included a message either encouraging voters to request a ballot because “[i]t’s safer for you to vote by mail!” or “[i]t’s safer for [neighborhood] to vote by mail!”^‡^ Fig. 1 illustrates sample postcards, which were mailed 18 May. The analyses below primarily consider these two postcard variants to be a single treatment.

We used a stratified, blocked randomization to determine who received postcards. We blocked on several variables that were measured before treatments were administered in the initial 2019 experiment as well as whether a registrant received postcards in the initial experiment.^§^ In all, 23,475 registered voters were assigned to the “self” condition and 23,485 to the “neighborhood” condition, with the remaining 888,785 in the control condition receiving no contact. We stratified treatment assignment such that approximately 50% of the 21,987 registrants who received four postcards in 2019 also received a postcard in 2020. Of these, 5,492 were assigned to the “neighborhood” message, 5,477 were assigned to the “self” condition, and the remaining voters were assigned to control (12). We used *t* tests to confirm that there were no significant imbalances in any of the 78 covariates used in the blocking, including subjects’ ward of residence, party registration, or prior vote history.^¶^

Mail ballots were sent only to those requesting them by the 26 May deadline. Mail ballots originally had to be received by the primary, but a last-minute policy change allowed ballots to be counted if they were postmarked by 2 June if received by 9 June.

## Outcomes and Results

*SI Appendix* provides additional information about our outcomes, which include whether subjects requested a mail ballot, failed to return a mail ballot, voted by mail, voted in person, or cast a provisional ballot, as well as when mail ballots were received.

**Fig. 2.**
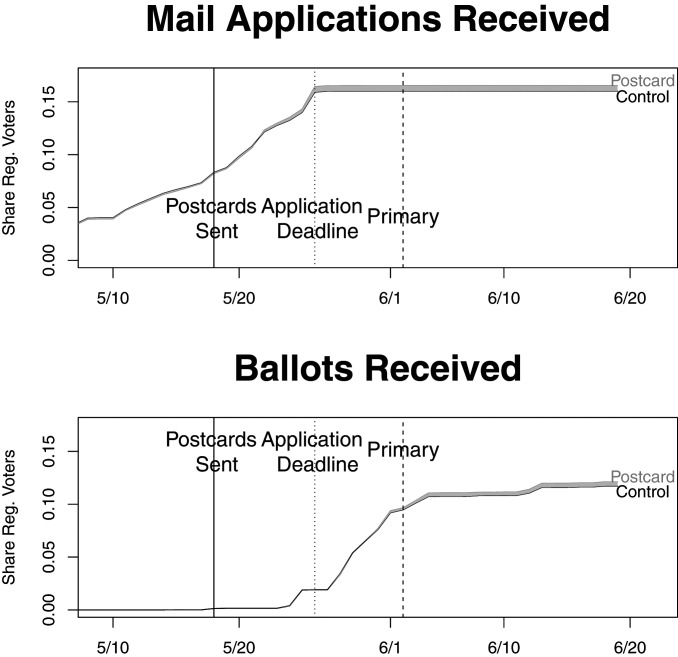
Share of voters who had applied for mail ballots, returned mail ballots by day.

In Fig. 2, we plot the fraction of our sample whose mail ballot application was received by the City Commissioners (Fig. 2, *Top*) and whose ballot was received (Fig. 2, *Bottom*). As expected, there was no difference in either applications received or mail ballots received before the postcards were mailed. However, those assigned to receive postcards subsequently applied for mail ballots and returned those ballots at higher rates.

How large are such differences? Table 1 presents *t* tests comparing those receiving a postcard to those not. The first column provides the full-sample results. Those assigned to treatment were 0.5 percentage points more likely to apply for a mail ballot (*P* = 0.004) and 0.4 percentage points more likely to vote by mail (*P* = 0.017). They were 0.2 percentage points more likely to have their ballot received after the primary and for it to be recorded (*P* = 0.01 for both). There was no significant impact on in-person voting (*P* = 0.462). While turnout overall was up 0.2 percentage points in the treated group, this difference is insignificant (*P* = 0.259). Together, the treatment group’s (insignificant) 0.13 percentage point decrease in in-person voting coupled with its (significant) 0.37 percentage point increase in mail voting suggest that roughly 35% (0.13/0.37) of the increased mail voting comes from substitution by people who would have otherwise voted in person.

**Table 1.** Means and *P* values from two-sided *t* tests comparing registered voters assigned to a postcard (T) to the control group (C)

		Did not		
		apply by	Pr(White)	Pr(Black)
	All	18 May	>0.5	>0.5
C: Requested mail ballot	0.160	0.084	0.192	0.166
T: Requested mail ballot	0.165	0.091	0.197	0.171
*P* value	0.004	0.000	0.124	0.052
C: Requested/no return	0.040	0.028	0.040	0.044
T: Requested/no return	0.041	0.030	0.041	0.045
*P* value	0.146	0.029	0.729	0.665
C: Voted by mail	0.120	0.056	0.152	0.122
T: Voted by mail	0.124	0.061	0.156	0.127
*P* value	0.017	0.000	0.135	0.051
C: Received by 2 Jun	0.095	0.037	0.124	0.094
T: Received by 2 Jun	0.096	0.040	0.127	0.096
*P* value	0.213	0.005	0.357	0.267
C: Received after 2 Jun	0.025	0.019	0.028	0.028
T: Received after 2 Jun	0.027	0.021	0.030	0.031
*P* value	0.010	0.005	0.162	0.061
C: Voted in person	0.166	0.178	0.137	0.219
T: Voted in person	0.165	0.176	0.138	0.215
*P* value	0.462	0.400	0.874	0.153
C: Provisional ballot	0.006	0.005	0.003	0.009
T: Provisional ballot	0.005	0.004	0.002	0.008
*P* value	0.149	0.215	0.142	0.214
C: Voted any method	0.287	0.234	0.289	0.341
T: Voted any method	0.289	0.237	0.294	0.342
*P* value	0.259	0.138	0.184	0.915
*n*	935,745	857,974	290,948	421,149

The results are essentially identical when reestimated via linear models conditioning on the variables included in the blocking.^#^ Note that the 2019 mailings had no effect on 2020 mail balloting, with a coefficient of −0.116 percentage points (SE = 0.220).^∥^ There is no detectable difference between the “self” and “neighborhood” messages (P=0.78), explaining why they are combined in most analyses.

In columns 2 through 4 of Table 1, we present the same results for subsets of the respondents who 1) had not already applied to vote by mail as of 18 May, 2) were identified based on their last name and census block group as more likely to identify as White, or 3) were similarly identified as more likely to identify as Black.** While these analyses were proposed after the completion of the experiment, and so are exploratory, they are well-motivated. The 77,771 people who applied for a mail ballot prior to receiving the postcard cannot have been influenced by it, so removing these respondents should reduce variance without inducing bias. Moreover, given long-standing concerns about racial inequalities in turnout, it is valuable to identify any dis parate effects across select racial groups (14, 15). We do not see substantively meaningful differences between respondents by imputed race.^††^ However, when removing those who had already applied for a mail ballot, some effects are estimated with more precision. For example, the effect on voting by mail increases by 27% to 0.5 percentage points, with P<0.0001. Also, the effect of returning a ballot by the primary becomes stronger (0.3 percentage points) and significant (*P* = 0.005). The effect of substitution from in-person voting remains similar, too.

## Conclusion

In Philadelphia’s June primary, encouraging voting by mail increased recorded votes by mail. Under certain conditions, mail ballots can certainly increase the use of the franchise. However, the procedures for counting mail ballots condition their ability to enfranchise. Mail ballots are at greater risk than ballots cast in person to be not counted because of clerical errors or procedural violations. For example, in Pennsylvania’s November 2020 election, thousands of mail ballots were not counted because they were not enclosed in secrecy envelopes, a provision not enforced in the primary. Also, it is plausible that primary voters were more knowledgeable about vote-by-mail options and that a similar information campaign in a highly salient general election could produce larger effects. In such conditions, political parties may well intervene to assist voters in navigating this process, too.

In the run-up to the 2020 elections, officials made substantial efforts through multiple media to educate voters about voting by mail. Our results provide an estimate of the impact of one such effort and may inform future efforts.^‡‡^ When summarizing existing research, ref. 16 concludes there is no evidence that factual direct mail informing recipients of an upcoming election increases turnout. However, we show that some voting-relevant factual information can be delivered via postcards. Indeed, the increased mail-ballot usage among recipients of our postcards is comparable to ref. 16’s estimate of the typical turnout increase from mail invoking civic duty.

## Supplementary Material

Supplementary File

## Data Availability

Anonymized R-compatible data have been deposited in Harvard Dataverse (https://doi.org/10.7910/DVN/HUUEGI).

## Footnotes

*This means no one who registered to vote after spring 2019 is included in the universe for this experiment. About 36% of individuals who registered to vote after spring 2019 voted by mail in the 2020 primary, as compared to just 12% of registrants in our experimental universe.

^†^The *P* value from the coefficient for being previously assigned to receive postcards in a regression predicting attrition is 0.95.

^‡^The Spanish read “¡Es más seguro votar por correo!” or “¡Es más seguro para [neighborhood] votar por correo!”

^§^The variables we blocked on were vote history, gender, party affiliation, and ward, all as measured in the 2019 voter file.

^¶^The vote history categories are described in *SI Appendix*.

^#^For example, the treatment effect for voting by mail is estimated via regression to be 0.38 percentage points (SE = 0.15, *P* = 0.012).

^∥^Nor is there evidence of an interaction between the 2019 and 2020 treatments from a separate model (0.24 percentage points, SE = 4.44 percentage points).

**We use ref. 13’s method to estimate the probabilities that a registrant is White or Black based on their last name and the racial composition of their census block group.

^††^Nor do we find messaging effects when looking at subjects by gender.

^‡‡^Future researchers may also use similar efforts as an instrument to study the downstream effects of voting by mail.
