# Supplementary material for: Results from a 2020 field experiment encouraging voting by mail
Source: Proc Natl Acad Sci U S A. 2021 Jan 19;118(4):e2021022118. doi: 10.1073/pnas.2021022118 (PMC7848624; doi:10.1073/pnas.2021022118)
Supplement: Supplementary File [file pnas.2021022118.sapp.pdf]

PNAS
www.pnas.org

# **Supplementary Information for**
# Results from a 2020 Field Experiment Encouraging Voting by Mail

Daniel J. Hopkins, Marc Meredith, Anjali Chainani, Nathaniel Olin, and Tiffany Tse.

Corresponding author: Daniel J. Hopkins
Email: danhop@sas.upenn.edu

**This PDF file includes:**

Supplementary text

**Supplementary Information Text**

**Vote History.** We classified voters into bins based on their vote history. The 28% of voters who voted in the 2015 or 2017 municipal primaries were first clustered as “municipal primary voters.” A second group was comprised of the 7% of registrants who were not municipal primary voters but voted in the 2015 or 2017 municipal elections. A third group (11%) did not meet the criteria for the first two but had voted in a 2016 or 2018 state/federal primary. A fourth group (29%) had a history of turning out only in the 2016 or 2018 general elections. Finally, the 25% who had participated in none of those elections were classified as “low turnout voters.”

**Vote Outcomes.** Our outcome measures are constructed as follows:

- Requested mail ballot—generated from data on mail ballots provided by Philadelphia City Commissioners; 16.0% of sample. (We designate people as having voted by mail if they are listed in the voter file as having voted either by mail or absentee.)
- Voted by mail in 2020 primary—generated from voter file's “vote method” field, with people designated as having voted by mail if they are listed as voting by mail or absentee; 12.0% of sample. (There were 96 voters listed in the voter file as voting by mail or absentee who did not appear in the City Commissioner's ballot request data; these individuals' ballots may have been rejected, and we classify these individuals as *not* having voted by mail. We also classify 1,079 voters who returned ballots by mail and whose ballots were recorded as per the mail ballot log but who were not listed as mail voters in the voter file as having voted to be consistent with the voter file.)
- Voted in 2020 primary—generated from voter file; 28.7% of sample.
